# In Vitro Antiviral Activity of Green Tea Polyphenon-60 against Avian Paramyxoviruses

**DOI:** 10.1155/2021/3411525

**Published:** 2021-12-06

**Authors:** Hang Minh Pham

**Affiliations:** Epidemiology and Pathology Department, National Institute of Veterinary Research, 86 Truongchinh, Dongda, Hanoi 10000, Vietnam

## Abstract

Avian paramyxoviruses (APMVs) have caused an economically significant drop in global domestic poultry production because of their high morbidity and mortality rates. Polyphenols are the major components of green tea that have great antiviral effects. This study aimed to evaluate the anti-APMV activities of polyphenon-60. Twelve APMV-1 strains representing three different pathotypes, two strains of APMV-2, one strain of APMV-3, and one strain of APMV-7 were propagated in chicken embryos. To determine the cytotoxic effect, chicken embryo fibroblasts were treated with the test compound in various concentrations. To assess the antiviral properties, time-dependent, dose-dependent, and virulence-dependent experiments were conducted in both cell and chicken embryo models. A reduction in virus titers was measured by the hemagglutination test. The inhibitory effect on virus adsorption to the chicken red blood cell (RBC) surface was examined by the hemagglutination inhibition test. The results showed that lentogenic and mesogenic APMV-1 strains, APMV-3 strain, and APMV-7 strain were significantly inhibited (*p* < 0.05) by polyphenon-60 at 50 *μ*g/ml, while the 50% cytotoxic concentration of the compound was 345 *μ*g/ml. Polyphenon-60 also exhibited the inhibitory activity against hemagglutination by NDV. Taken together, the results suggest that polyphenon-60 has shown promise as an antiviral agent that has wide safety margins against APMVs, and challenge studies to evaluate its efficacy in chickens are necessary.

## 1. Introduction

Avian paramyxoviruses (APMVs) have been detected from a wide variety of avian species worldwide with variable clinical outcomes and economic impacts [1]. Newcastle disease virus (NDV), formally termed avian paramyxovirus serotype-1 (APMV-1), is the one of the twenty-one serotypes that has been officially recognized [2]. NDV strains can be differentiated based on their pathogenicity for chicken inoculation. Highly virulent (velogenic) strains cause acute infections with high mortality, bilateral conjunctivitis, preceded by respiratory and neurological signs, or hemorrhagic lesions in the gut [3]. The intracerebral pathogenicity index (ICPI) values for velogenic strains are greater than 1.5. Moderately virulent (mesogenic) strains with ICPI values ranging from 0.7 to 1.5 cause slight depression, lower mortality rates, and decrease egg production in laying flocks. Low virulent or nonpathogenic (lentogenic) strains may produce only mild respiratory signs in young chickens with very low mortality rates, low reduction in egg production, and ICPI values of less than 0.7 [4].

APMVs are enveloped with a single-stranded, nonsegmented, negative-sense RNA genome ranging from 14.9 to 17.4 kb and encode at least six proteins: nucleocapsid (N), phosphoprotein (P), matrix (M), fusion (F), hemagglutinin-neuraminidase (HN), and large polymerase (L) [5]. NDV infection is initiated by receptor recognition and binding to the host cell surface, which is followed by fusion mediated by the combined action of the HN protein and the F protein [6, 7]. Despite the fact that vaccination programs provided significant protection against ND outbreaks, the virus remained a potential threat to commercial poultry operations as well as backyard producers due to mutations in virus leading to reduce vaccine efficacy [8]. Thus, natural medicinal products have been considered a complementary eradication measure of this disease in developing countries.

Polyphenols are the major components of green tea that have attracted significant attention in the scientific and consumer community for their health benefits. Catechin (C), epigallocatechin-3-gallate (EGCG), epicatechin gallate (ECG), epigallocatechin (EGC), and epicatechin (EC) are the active ingredients of polyphenols [9]. Various biological and pharmacological activities have used the green tea polyphenols including antioxidant, antitumor, antibacterial, antiviral, and antiparasite activities [9–16]. Many previous studies have reported that the mechanisms of antiviral activity of green tea polyphenols, especially EGCG, involve the stages of viral attachment and genome synthesis [9]. However, the inhibitory effects of green tea polyphenols on the growth of NDV as well as other APMV serotypes have not yet been demonstrated.

Therefore, this study aimed to evaluate the anti-APMV activities of green tea polyphenols (polyphenon-60).

## 2. Materials and Methods

### 2.1. Cell Culture, Viruses, and Compounds

Chicken embryo fibroblast (CEF) cells were cultured in Dulbecco's Modified Eagle Medium (DMEM) (Gibco, Invitrogen) supplemented with 10% fetal calf serum (FCS), 100 IU/ml penicillin, 100 mg/ml streptomycin, and 2 mM L-glutamine at 37°C in a humidified atmosphere containing 5% CO_2_. APMV serotypes are listed in Table 1; all were reported previously [17] and were propagated in the allantoic cavity of 10-day-old chicken embryos for making viral stocks. The allantoic fluids were harvested, tested for hemagglutination (HA) activity, and then kept frozen at −80°C until use. Polyphenon-60 extracted from green tea (Sigma Chemical, St. Louis, Mo.) was used in all experiments. The stock solution of 1 mg/ml was prepared in deionized water and was sterilized by passage through a 0.22 *μ*m-pore-size syringe tip filter. The solution was then solubilized in the tissue culture medium for in vitro experiments or in distilled water for in vivo studies.

### 2.2. Hemagglutinin (HA) and Hemagglutination Inhibition (HI) Assays

The HA assay was conducted to measure the reduction in virus titers. Each well on a V-bottom 96-well plate was dispensed with 25 *μ*l of PBS. Subsequently, 25 *μ*l of the supernatant of infected cells or the infected embryonic fluids was added to each well of the first row on the plate. Two-fold serial dilutions were carried out until the second last well on the plate. The last well was used as a control well and was filled with PBS. Then, 25 *μ*l of 0.5% chicken red blood cell (RBC) suspension was added to each well, and the plate was allowed to keep at room temperature for 45 min. A negative result displayed as a red point at the bottom center of the well, and a positive result was the formation of a diffuse mat on the bottom of the well. The virus titers were displayed as HA units and were determined as the inverse of the virus last dilution showing complete agglutination.

The HI assay was conducted to examine the inhibitory effect of polyphenon-60 on virus adsorption to the chicken RBC surface and was performed as described for NDV [18]. In brief, 25 *μ*l (4 HAU) NDV was mixed with 25 *μ*l of polyphenon-60 at different concentrations in a V-bottomed 96-well plate at room temperature for 30 min. This mixture was subsequently incubated with 50 *μ*l of 0.5% chicken RBCs at room temperature for 45 min. The inhibition hemagglutination of polyphenon-60 was observed after the incubation period. The virus in the absence of polyphenon-60 was used as a negative control, and PBS was used as a positive control.

### 2.3. 50% Tissue Culture Infectious Dose and 50% Embryo Infectious Dose

PBS was used to prepare eight 10-fold serial dilutions of a viral stock. The TCID_50_ assay was performed using the confluent CEF monolayers. Each well of a 96-well flat-bottom plate was inoculated with 20 *μ*l dilution. After 1 hour for viral infection, the supernatant was replaced with 100 *μ*l of DMEM containing 5% FBS. To monitor the cytopathic effect (CPE), the plate was incubated for 5 days at 37°C. The EID_50_ assay was performed using 9-day-old SPF chicken embryos. Each of the five embryonic eggs was inoculated with 0.1 ml each dilution and incubated for four days. After the incubation period, all eggs were tested for the presence of HA activity. The TCID_50_ and EID_50_ titrations were calculated using the Reed–Muench method [19].

### 2.4. Cytotoxicity Assay

The cytotoxic concentration (CC) of polyphenon-60 was evaluated using the alamarBlue assay. Each well of the subconfluent monolayer CEF cell on a flat-bottom 96-well plate was washed three times with PBS and treated with 100 *μ*l of serum-free medium with varying concentrations of polyphenon-60 or left untreated for the control. Each concentration was performed three times. The cells were incubated at 37°C for 48 hours to allow for proliferation and were then stained with 10% alamarBlue (Serotec, Oxon, UK) for 4 hours at 37°C in the dark. Reduction of alamarBlue was measured at wavelengths of 562 nm and 595 nm in a microplate reader MTP-650FA (Corona Electric, Hitachinaka, Japan). The percentage reduction (PR) was calculated using the manufacturer's formula: [A_LW_ − (A_HW_ × R_0_) for test well/A_LW_ − (A_HW_ × R_0_) for the control well] × 100. The correction factor (R_0_) was calculated with the following formula: R_0_ = AO_LW_/AO_HW_. The CC_50_ value was determined using linear regression analysis.

### 2.5. Dose- and Virulence-Dependent Assays

Confluent CEF monolayers on a 48-well flat-bottom plate were infected with a strain at 100 TCID_50_ and were incubated for 1 hour at 37°C. The cells were washed three times with PBS for removing nonabsorbed virus and were treated with the medium containing the desired concentration of polyphenon-60. Each concentration was performed in triplicate. The induction of CPE in the host cells was observed daily. After 48 hours of incubation, the alamarBlue assay was performed as described above. A well without virus served as a positive control indicating 100% inhibition of CPE. A well with viral infection served as a negative control indicating 0% inhibition. The percent inhibition of CPE was calculated using the following formula: (PR value_drug treated_ − PR value_negative control_)/(PR value_positive control_ − PR value_negative control_) × 100 [20].

### 2.6. In Vitro Antiviral Activity

Twelve APMV-1 strains representing three different pathotypes, two strains of APMV-2, one strain of APMV-3, and one strain of APMV-7 were propagated in chicken embryos. Confluent CEF monolayers on a 96-well flat-bottom plate were inoculated with a virus strain at 4 HA units (HAU)/25 *μ*l and were incubated at 37°C for 1 h. After washing three times with PBS, the cells were overlaid with the medium containing varied concentrations of polyphenon-60 and incubated for 48 hours. The supernatants were harvested, and the viral titers were determined by the HA assay.

### 2.7. In Ovo Antiviral Activity

Each concentration (20 *μ*g/ml and 50 *μ*/ml) of polyphenon-60 was mixed with an equal volume of the NDV at 100 EID_50_. Each of the virus-compound mixtures was then inoculated into five 10-day-old chicken embryos. A negative embryo was incubated with the virus and PBS. A positive embryo was incubated with PBS only. Embryo viability was examined daily. Embryo death was recorded as the signs of a breakdown of visible blood vessels of the CAM, blood leakage into the yolk or the allantoic fluid areas, the appearance of disseminated coagulation, and the absence of embryonic movement, and the egg was refrigerated overnight. Up to 24 hours, any dead embryo was discarded. Each allantoic fluid was harvested, and the presence of NDV in the allantoic fluid was tested by the HA assay. The survival rates of embryos were recorded on day 5 (120 h) after inoculation.

### 2.8. Time-of-Addition Assay

A time-of-addition assay was conducted to investigate the antiviral effects of polyphenon-60 on different stages of the viral infection. Confluent CEF monolayers in a flat-bottom 96-well plate were infected with the NDV B1 strain at 100 TCID_50_. Polyphenon-60 at 50 *μ*g/ml was added to the cells in different time intervals: at 1 hour prior to infection (preinfection), at the time of infection (infection), and 1 hour, 6 hours, and 12 hours after viral infection (postinfection). The virus-compound mixture was incubated for 48 hours at 37°C. The inhibition effect of polyphenon-60 was evaluated using the alamarBlue assay as described earlier.

### 2.9. Statistical Analysis

Data were expressed as the mean ± SD of three independent experiments. The differences between the means were analyzed by using Student's *t*-test. Statistical significance was considered as *p* values < 0.05.

## 3. Results

### 3.1. Cytotoxicity of Polyphenon-60

**Figure 1:**
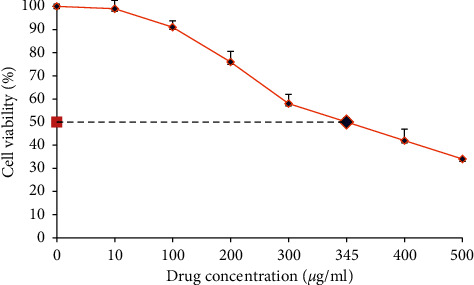
Viability of CEF cells (%) treated with different concentrations of polyphenon-60 for 48 h p.i. The dot line represented 50% cell viability. Data were expressed as the mean ± SD of three independent experiments.

The cytotoxicity of polyphenon-60 was performed using subconfluent monolayer CEF cells and was measured using the alamarBlue assay. At low concentrations, 10 *μ*g/ml to 100 *μ*g/ml showed the percentage of cell viability was more than 90%, while the concentrations above 400 *μ*g/ml led to reductions of more than 50% cell viability (Figure 1). The CC_50_ value estimated using regression analysis was 345 *μ*g/ml.

### 3.2. Antiviral Activity

**Figure 2:**
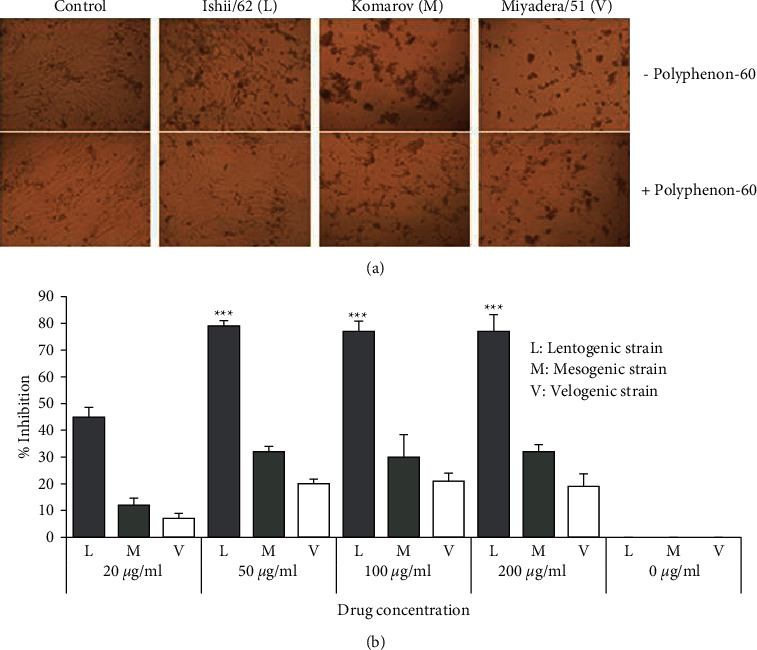
The inhibitory activity of polyphenon-60 on the infection of NDV. (a) The inhibitory activity of polyphenon-60 on cytopathic effects of NDV in CEF cells at 48 h p.i. (b) The antiviral activity of polyphenon-60 against different NDV pathotypes. Data were expressed as the mean ± SD of three independent experiments and compared by using Student's *t*-tests. ^*∗∗∗*^Highly statistically significant (*p* < 0.001).

NDV strains with three different pathotypes included velogenic strain (NDV/Miyadera/51), mesogenic strain (NDV/Komarov/40), and lentogenic strain (NDV/Ishii/62) at 100 TCID_50_, and polyphenon-60 at different concentrations (0 *μ*g/ml, 20 *μ*g/ml, 50 *μ*g/ml, 100 *μ*g/ml, and 200 *μ*g/ml) was prepared. The antiviral activity was evaluated using the dose- and virulence-dependent assays and was measured using the alamarBlue assay. The CEF cells infected with NDV in the presence or absence of polyphenon-60 showed a differential reduction of virus-induced CPE (Figure 2(a)). The mesogenic and velogenic virus-infected cells were treated with the compound at 50 *μ*g/ml which showed less extensive CPE than those untreated cells. The lentogenic virus-infected cells were treated with the same concentration showing cell morphology similar to control cells (uninfected cells). The highest percentage inhibited by the lentogenic strain was 79% at the concentration of 50 *μ*g/ml. At the lowest concentration of 20 *μ*g/ml, the inhibition ratio was less than 50%. Significant inhibition ratios were also found in the infected cells treated with polyphenon-60 at concentrations of 100 *μ*g/ml and 200 *μ*g/ml. However, in the same concentrations, the maximum inhibition ratios of mesogenic and velogenic strains were only ≈32% and 20%, respectively (Figure 2(b)).

To further investigate whether polyphenon-60 could prevent other APMV replications, CEF cells were inoculated with APMV type-1 representing three pathotypes (velogenic, mesogenic, and lentogenic), -2, -3, and -7 at 8 HA units (HAU)/50 *μ*l and were treated with different concentrations at 0 mg/ml, 20 mg/ml, 50 mg/ml, and 100 mg/ml. High doses of polyphenon-60 exhibited a significant reduction of viral HA titers (log_2_ HAU) in the cells infected with two lentogenic strains (NDV/Hitcher B1/48 and NDV/Ishii/62), three mesogenic strains (NDV/Komarov/40, NDV/Tochigi/95, and NDV/Kumamoto/2000), and APMV-3 and APMV-7 strains. The same doses showed a light reduction of viral HA titers in the cells infected with velogenic (NDV/Sato/30 and NDV/Taka/73) and two APMV-2 strains. However, the viral HA titers of the cells infected with velogenic NDV strains (NDV/Niigata/85, NDV/Tokyo/96, NDV/Ibaraki-2/99, and NDV/Chiba/2001) were similar to those in untreated cells (Table 1).

### 3.3. In Ovo Antiviral Activity

To expand on the finding, the egg models were used to evaluate the antiviral activity of polyphenon-60. The results showed that polyphenon-60 extended the time and increased the rate of embryo survival. All untreated embryos that were infected with the velogenic (NDV/Miyadera/51) strain died within 48 hours. The percent survival at 60 and 72 hours after treatment was only 20%. The embryos infected with the mesogenic (NDV/Komarov/40) strain did not survive beyond 72 and 84 hours after treatment. In contrast, all embryos infected with the lentogenic (NDV/Ishii/62) strain and treated with 50 *μ*g/ml of polyphenon-60 survived until day 5 (120 hours) after inoculation (Table 2).

### 3.4. Time-Dependent Inhibitory Effects

**Figure 3:**
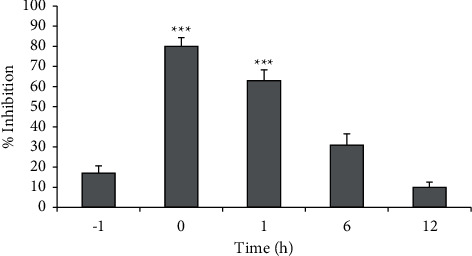
Time-dependent inhibition of polyphenon-60 on the replication cycle of the NDV B1 strain. Data were expressed as the mean ± SD of three independent experiments and compared by using Student's *t*-tests. ^*∗∗∗*^Highly statistically significant (*p* < 0.001).

In order to determine the stage of the NDV life cycle blocked by polyphenon-60, the time-of-addition assay was conducted. As shown in Figure 3, the highest inhibition rate of the compound was 80% at 0 h p.i. The inhibition rates reduced to less than 50% when polyphenon-60 was added at 6 h p.i and 12 h p.i. In pretreated cells, the compound's inhibition rate was only 17%. These results suggested that polyphenon-60 affected the early stages of the virus life cycle.

### 3.5. Hemagglutination Inhibition of Polyphenon-60

**Figure 4:**
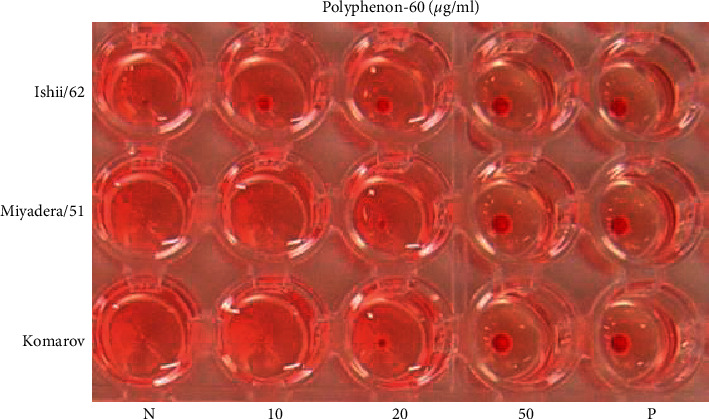
Inhibitory effect of polyphenon-60 on the hemagglutination of NDV strains. 4 HAU NDV strains with three different pathotypes were incubated with polyphenon-60 at different concentrations (10 *μ*g/ml, 20 *μ*g/ml, and 50 *μ*g/ml). The well with untreated virus served as the negative control (N), and the well with only PBS served as the positive control (P). Three independent experiments were performed.

To determine the inhibitory effect of polyphenon-60 on virus adsorption to the chicken RBC surface, the HI test was performed. At all concentration tests, polyphenon-60 completely inhibited HA activity of the NDV/Ishii/62 (lentogenic) strain. No hemagglutination occurred after polyphenon-60 at the highest concentration (50 *μ*g/ml) was mixed with the NDV/Komarov/40 (mesogenic) and the NDV/Miyadera/51 (velogenic) strains (Figure 4). These results suggested that polyphenon-60 might attach to the virus and prevent the virus-RBC binding that inhibits hemagglutination.

## 4. Discussion

APMV infections continue to emerge as a disease threat in both turkeys and chickens [21]. The options for preventing AMPV transmission included applying adequate biosecurity measures, placing restrictions on movements of birds, quarantining suspected flock, culling infected birds, and using vaccinations. However, backyard poultry production systems with low biosecurity measures, deficient vaccines, antigenic variation, the short duration of the immune response, and immune suppression resulted in the persistence of virulent NDV in the flock and the environment [22]. Therefore, the development of effective antiviral compounds combined with a vaccination program has a positive impact on the prevention and control of APMV.

Many previous studies used epigallocatechin-3-gallate (EGCG), which was a major polyphenol of green tea, against various human and animal pathogen viruses. At a concentration of 50 *μ*M, EGCG efficiently inhibited hepatitis B virus (HBV) replication by inducing a complete autophagic process [23]. At the same concentration, EGCG inhibited hepatitis C virus (HCV) infection by more than 90% at an early stage of the viral life cycle [20]. EGCG at higher concentrations (≥100 *μ*M) reduced ≥1,000-fold herpes simplex virus type 2 (HSV-2) titers in 10 to 20 min and reduced the same amount of HSV type 1 (HSV-1) titers in 30 to 40 min [24]. Catechins (195 *μ*g/ml) were able to completely inhibit the synthesis of SARS-CoV antigens [25]. At a concentration of 10 g/kg feed of green tea, byproducts exhibited a significant inhibitory activity (*p* < 0.001) against the H9N2 avian influenza virus (AIV) in chickens [26]. EGCG (50 *μ*M) combined zinc sulfate and silver nanoparticles which exhibited very strong antiviral activity (*p* < 0.001) against the H5N1 AIV, with a reduction in the log titer of the virus by up to 7.6 times [27]. The combination of EGCG (25 *μ*M) and zinc sulfate exhibited strong antiviral activity (*p* < 0.01) against peste des petits ruminants virus (PPRV) [28]. Wang et al. [29] found that tea polyphenols (TPPs) suppressed PRRSV load in the stages of viral attachment, internalization, replication, and release. However, none of these studies have used tea polyphenols for antiviral activity against APMV.

In this study, tea polyphenols demonstrated the inhibitory activity against NDV infection in CEF cells and chicken embryos. The concentrations, virulent strains, and drug addition times influenced the degree of compound inhibition. When polyphenon-60 was treated with cells infected with a lentogenic strain at a concentration of 50 g/ml at 0 h, significant inhibition of polyphenon-60 was observed (*p* < 0.001). Polyphenon-60 also significantly reduced (*p* < 0.05) the HA titration of two lentogenic and three mesogenic NDV strains, APMV-3, and APMV-7. In in ovo experiments, polyphenon-60 at 50 *μ*g/ml prolonged the survival period of embryos infected with mesogenic and velogenic strains. At the same concentration, it completely protected the embryos infected with the lentogenic strain from death. However, polyphenon-60 showed no inhibitory effect on four velogenic NDV strains (NDV/Niigata/85, NDV/Tokyo/96, NDV/Ibaraki-2/99, and NDV/Chiba/2001) when virucidal activity was not observed at any concentration of the compound.

Although the HA assay has been used for the quantification of viral infectious units in many studies [30–32], its sensitivity is severely limited when it is compared to real-time PCR assay [33]. The limitation of this study was using the HA assay to quantify the reduction in virus titers. However, APMV strains with four different serotypes (APMV-1, APMV-2, APMV-3, and APMV-7) and three different pathotypes (velogenic, mesogenic, and lentogenic) were tested; the HA assay was a more simple and lower-cost assay to produce the results than the real-time PCR assay. The results of the HA quantification test of 10 NDV strains could be used to compare within-group and between-groups. The results of a real-time quantitative PCR test for a single strain were used for the comparison of between-groups only. These were the reasons why the HA assay was used rather than using the real-time PCR assay in this study.

Through mediating membrane fusion, the fusion (F) protein of NDV could affect viral infection and pathogenicity [6]. The antiviral activity of polyphenon-60 was widely different among the three pathotypes of NDV. The compound exhibited strong inhibition activity against the replication of the lentogenic strain. It exhibited moderate to very weak inhibition activity against the replication of mesogenic and velogenic strains. These results suggested that polyphenon-60 inhibited virus-cell fusion by interacting with the F0 protein (avirulent strain) but not with the F protein (virulent strain). Similar results were obtained when Vero cells were infected with the avirulent NDV (LaSota) strain and were treated with fucoidan [34]. Slight inhibition was observed when CEF cells were pretreated (−1 h) with the compound before the infection of NDV. The deposition of this compound on monolayer cells might prevent viral adsorption. NDV bound to chicken RBCs through the HN binding site [35]. The results of the hemagglutination inhibition assay suggested that polyphenon-60 might bind to NDV, thereby inhibiting the HA activity of the virus.

For thousands of years, tea consumption indicates a lower risk of the toxicity in tea and tea polyphenols [36]. In this study, polyphenon-60 did not cause significant damage to CEF cells at any concentration less than 350 *μ*g/ml, while the antiviral effective doses of the compound ranged from 50 *μ*g/ml to 200 *μ*g/ml. Polyphenon-60 was found to have wide safety margins.

The in ovo application of various materials in birds included vaccines, drugs, hormones, probiotics and prebiotics, peptides, carbohydrates, vitamins, and plant extracts [37]. Conventional supplementation routes such as in-feed and in-water were less economical routes than in ovo delivery of bioactive substances [38]. Moreover, many studies have used the in ovo model for the evaluation of the antiviral efficacy of herbal extracts instead of using the chicken model [27, 39, 40]. The chicken embryo is an inexpensive and efficient screening model for drug discovery [41] and a well-available in vivo animal model in the field of scientific experimentation and application [42]. However, discrepancy between the results of in ovo and chicken models might sometimes occur such as dsRNA had antiviral activity in ovo and in vitro but not in chickens after hatch [40]. The differences in routes of administration and the age of the animals might have contributed to this discrepancy. The results of this study showed the antiviral effects of polyphenon-60 in in ovo and in vitro models. Future clinical trials with chickens are necessary to support the effective use of polyphenon-60 as one medicament for poultry.

## 5. Conclusion

Considering newly emerging NDV and other APMVs, the use of green tea polyphenon-60 will be useful for inhibiting virus infection. The results presented in this study suggest that polyphenon-60 with inhibitory effects against APMV in in vitro and in ovo models might be developed as a potential antiviral agent that has wide safety margins. In the future, challenge studies to evaluate its efficacy in chickens will be explored.

## Figures and Tables

**Table 1:** Inhibitory effect of polyphenon-60 on the growth of avian paramyxoviruses in CEF cells.

Strain	Virulence^*∗*^	The virus infectivity titers at 48 h p.i in the presence of polyphenon-60 (log_2_ HAU)			
		100 (*μ*g/ml)	50 (*μ*g/ml)	20 (*μ*g/ml)	0 (*μ*g/ml)

NDV/Sato/30	V	2.3 ± 0.6	2.3 ± 1.2	2.7 ± 1.2	3.3 ± 0.6
NDV/Komarov/40	M	3.3 ± 0.6^a^	2.7 ± 0.6^a^	4.3 ± 0.6^b^	4.7 ± 0.6^b^
NDV/Hitcher B1/48	L	1.7 ± 1.2^a^	1.7 ± 0.6^a^	4.3 ± 0.6^b^	5.3 ± 0.6^b^
NDV/Miyadera/51	V	2.3 ± 1.5	2.3 ± 1.2	2.7 ± 0.6	3.0 ± 1.0
NDV/Ishii/62	L	1.3 ± 0.6^a^	1.7 ± 0.6^a^	4.3 ± 0.6^b^	5.3 ± 0.6^b^
NDV/Taka/73	V	2.0 ± 1.0	2.0 ± 0.0	2.7 ± 0.6	2.7 ± 0.6
NDV/Niigata/85	V	3.0 ± 1.0	3.0 ± 0.0	3.0 ± 1.0	3.0 ± 1.7
NDV/Tochigi/95	M	2.7 ± 0.6^ab^	3.7 ± 0.6^b^	4.3 ± 0.6^b^	4.7 ± 0.6^b^
NDV/Tokyo/96	V	2.7 ± 0.6	2.7 ± 1.0	2.7 ± 0.6	2.7 ± 1.0
NDV/Ibaraki-2/99	V	2.0 ± 0.0	2.0 ± 1.0	2.3 ± 0.6	2.0 ± 1.0
NDV/Kumamoto/2000	M	3.0 ± 1.0^b^	2.3 ± 0.6^ab^	4.0 ± 1.0^b^	4.7 ± 0.6^b^
NDV/Chiba/2001	V	2.7 ± 0.6	2.7 ± 1.2	2.7 ± 0.6	2.7 ± 1.5
APMV-2/Yucaipa/56	NA	2.3 ± 1.2	2.7 ± 0.6	2.7 ± 0.6	2.7 ± 0.6
APMV-2/Bangor/73	NA	2.0 ± 1.0	2.3 ± 0.6	2.7 ± 0.6	2.7 ± 1.5
APMV-3/Winsconsin/68	NA	3.7 ± 0.6^a^	3.3 ± 0.6^a^	5.3 ± 0.6^b^	5.7 ± 0.6^b^
APMV-7/Tennessee/75	NA	1.0 ± 0.0^ab^	1.7 ± 1.2^b^	2.0 ± 1.0^b^	2.3 ± 0.6^b^

^*∗*^L: lentogenic; M: mesogenic; V: velogenic; NA: not applicable. Data were expressed as the mean ± SD of three independent experiments. Means with different superscript letters in a row were significantly different (*p* < 0.05).

**Table 2:** Inhibitory effect of polyphenon-60 on the growth of NDV strains in chicken embryonated eggs.

NDV strain	Virulence^*∗*^	Concentration (*μ*g/ml)	Time of survival (h)	Number of live embryos	Survival rates (%)

Miyadera/51	V	Untreated	48	0	0
		20	60	1	20
		50	72	1	20

Komarov/40	M	Untreated	60	0	0
		20	68	1	20
		50	84	1	20

Ishii/62	L	Untreated	96	0	0
		20	120	1	20
		50	120	5	100

^*∗*^L: lentogenic; M: mesogenic; V: velogenic.

## Data Availability

All the research data used to support this study are included within the manuscript.
